# Making Landscapes Negotiable: Q-methodology as a Boundary-Spanning and Empowering Diagnostic

**DOI:** 10.1007/s00267-024-02004-1

**Published:** 2024-06-10

**Authors:** James Douglas Langston, Mirjam A. F. Ros-Tonen, James Reed

**Affiliations:** 1 CSIRO Environment, Building 101, Clunies Ross Street, Black Mountain, ACT 2601 Australia; 2 Faculty of Forestry, University of British Columbia, 2424 Main Mall, Vancouver, BC Canada; 3 Department of Geography, Planning and International Development Studies, University of Amsterdam, Amsterdam, The Netherlands; 4 Center for International Forestry Research (CIFOR), Bogor, Indonesia; 5 University of East Anglia School of Global Development, Norwich Research Park, UK

**Keywords:** Q-methodology, Integrated landscape approaches, Discourses, Imaginaries, Boundary tool

## Abstract

Landscapes are conceptually fuzzy and rich, and subject to plural framings. They are places of inquiry and intervention for scientists and practitioners, but also concepts bound to peoples’ dynamic identities, knowledge systems, inspiration, and well-being. These varying interpretations change the way landscapes function and evolve. Developed in the 1930s, Q-methodology is increasingly recognized for being useful in documenting and interrogating environmental discourses. Yet its application in the context of how integrated landscape approaches better navigate land-use dilemmas is still in its infancy. Based on our experience and emerging literature, such as the papers in this special collection, this article discusses the value of Q-methodology in addressing landscape sustainability issues. Q-methodology helps unravel and communicate common and contradicting landscape imaginaries and narratives in translational and boundary-spanning ways, thus bridging actors’ different understandings of problems and solutions and revealing common or differentiated entry points for negotiating trade-offs between competing land uses. The methodology can be empowering for marginalized people by uncovering their views and aspirational values to decision-makers and policymakers. We argue that this potential can be further strengthened by using Q to identify counter-hegemonic discourses and alliances that combat injustices regarding whose knowledge and visions count. In this way, applying Q-methodology in integrated landscape approaches can become a key tool for transitioning toward just, inclusive, and sustainable landscapes.

## Introduction

Landscape is a relational concept that integrates environmental and social dynamics (Antrop 2005; Drexler 2013; Stenseke 2018). For many peoples and cultures, landscapes represent holistic, spatially bound areas that hold certain values and meanings, strongly associated with a sense of place and culture. They are typically acknowledged as dynamic spaces that have been, and continue to be, shaped by—increasingly globally interconnected—interactions between natural, cultural, economic, technological, and political processes (Naveh 1995; Görg 2007; Liu et al. 2019; Munroe et al. 2019).

The use and management of landscapes are influenced by people’s intrinsic, instrumental, and relational values (Arias-Arévalo et al. 2017; Klain et al. 2017; Himes and Muraca 2018; Stenseke 2018). The weight attached to each of these values is often highly variable, depending on a range of factors, such as people’s professional background, personal characteristics like age and gender, experience, status, network, worldview, political persuasion, and objectives. Nevertheless, these values strongly determine the subsequent actions, activities, and institutions that shape landscapes. Hence, in the last few decades, global conservation and development communities collectively imagine landscapes as spaces where multiple interests intersect and interconnected challenges can be addressed. This implies recognition of trade-offs between potentially competing goals and land uses and the need for interventions that aim to reconcile social, ecological, and political objectives (Sayer 2009; Larson et al. 2021).

Landscape-scale interventions have subsequently proliferated, evolving from a focus on primarily biodiversity conservation to encompassing broader environmental, socio-cultural, political, commercial, and governance issues within, and increasingly beyond, a landscape of interest. These interventions have taken various guises, with more recent iterations tending to engage multiple actor groups in land use decision-making negotiations. Often described as *integrated landscape approaches* (ILAs) (Sayer et al. 2013; Reed et al. 2016, 2020b; Arts et al. 2017; Pedroza-Arceo et al. 2022) or a negotiated form of landscape governance (Ros-Tonen et al. 2021; Siangulube et al. 2023), they are conceived as long-term collaborative approaches to identify, reconcile and ideally satisfy multiple and competing interests. The ultimate aim is to ‘win more and lose less’ in terms of benefits and losses for nature and different actor groups (Sayer et al. 2013; Reed et al. 2015; Ros-Tonen et al. 2018).

However, landscape dilemmas are wicked problems (Scherr and McNeely 2008; Sayer et al. 2013; Freeman et al. 2015; Reed et al. 2016, 2020b), implying there is no single definition and explanation for such problems and no simple solutions (Rittel and Webber 1973). Every wicked problem is “essentially unique” (Ibid., p. 164) and is characterized by high levels of uncertainty and disagreement (Balint 2011). Hence, seeking change for improved landscapes requires governance mechanisms that recognize that “context is everything” (Sayer and Margules 2017; Langston et al. 2019a).

‘Landscape’ is a relational space shaped by biophysical characteristics and human behavior. Cultural and emotional attachment to landscapes interact with these characteristics (Arias-Arévalo et al. 2017; Klain et al. 2017; Himes and Muraca 2018; Stenseke 2018). This interconnectedness requires that landscape governance explicitly deals with contextualized issues, whether geographic, biophysical, political, socio-economic, or cultural (Minang et al. 2014; Reed et al. 2016; Pedroza-Arceo et al. 2022).^1^ Local-specific governance mechanisms are expected to manifest our capabilities to govern complexity more effectively, sustainably, and equitably (van Oosten et al. 2021). Understanding nature and people and the interrelated dynamics of both merits transdisciplinary, place-based learning mechanisms that are embedded among the actors influencing landscapes (Tress et al. 2001; Langston et al. 2019b).

Integrated landscape approaches are built upon assumptions that a diversity of people can develop and co-create aspirational change pathways (Reed et al. 2023). Doing so requires capturing diverse views and navigating trade-offs and potential conflicts (Amorim de Castro et al. 2024; Bayala 2024; Jayaprakash and Hickey 2024; Siangulube 2024, this issue). Therefore, clarifying the change logic and underlying perceptions and assumptions is imperative (Sayer et al. 2013; Reed et al. 2023). Traditional methods and learning mechanisms are often ill-equipped to meaningfully process the full range and complexity of ontologically diverse ways of interpreting landscape challenges and solutions.

Opportunities lie in methodologies that recognize, consider, and engage with how people imagine change in their landscapes (Nogué and Wilbrand 2018). Because these understandings are usually contested and seemingly irreconcilable, we are attracted to methods that unravel different landscape imaginaries and discourses as a basis for solving landscape dilemmas and do so in engaging ways. As shown in this special issue, there are several such methods, including participatory scenario building and spatially explicit simulation (Asante-Yeboah et al. 2024)^2^, fuzzy cognitive mapping (Badry et al. 2024), multi-actor dialog based on semi-quantitative ecosystem ranking (Pham-Truffert and Pfund 2024), systemic co-inquiry (Amorim de Castro et al. 2024), and Q-methodology (Ng et al. 2023; Bayala 2024; Dugasseh et al. 2024; Jayaprakash and Hickey 2024; Siangulube 2024). What these and other methods have in common are their interactive and semi-quantitative approaches toward unraveling multiple actor interests, values, and perspectives (Ros-Tonen et al. 2024).

Aside from the methods mentioned above, new and innovative landscape tools, such as immersive 3D visualization, virtual reality, and scenario-based tools that make use of artificial intelligence and landscape generation (e.g., Celio et al. 2015; Wang et al. 2016; Metze 2020), may also be attractive. However, they are less suitable and practical for tropical and poorly accessible or resourced contexts. Moreover, in line with calls to decolonize methodologies (and, by consequence, bridge ‘researcher—subject’ divides), methods should avoid exacerbating inequities along multi-dimensional power asymmetries, such as through access or application of advanced states of technology. As researchers, we should not presume that marginalized people will perceive the use of advanced technology as a welcome form of engagement for reflexivity, deliberation or envisioning (Williams 2004).

In light of these deliberations, this paper focuses on the relatively easy-to-apply and cost-effective Q-method. This method is gaining increasing attention in environmental governance and transdisciplinary research due to its potential to reveal divergent perspectives on sustainability and place-based issues in diverse human, economic and environmental geographies and contexts. As such, it helps governance systems deal with fundamental complexities. In this article, we contribute to the emerging literature on the Q-method by considering its usefulness for addressing landscape dilemmas.

## Landscape Imaginaries and Discourses

Contemplating what happens if we discuss landscapes beyond fairly homogenous groups or professional circles, Meinig (1979) virtually brought together a group of professionals to the same spot in a landscape, asking what they saw and what it meant to them. The exercise made clear that it was fairly easy to agree on “facts” such as the number of trees, the course of a river, or the form of houses. However, making sense of these “facts” revealed that landscape is a “contested term” and that “any landscape is composed not only of what lies before our eyes but what lies within our heads” (Meinig, 1979, p. 33-34). This helps reveal how facts do not speak for themselves but are framed by people and their values (Turnhout 2024). Identifying different interpretations among differently empowered actors is key to more just and effective communications—essential in landscape approaches that advocate landscape management based on stakeholder negotiations.

In recent literature, several authors frame these interpretations as “landscape imaginaries” (e.g., Shankland and Gonçalves 2016; Nogué and Wilbrand 2018; Walsh 2020). The term draws from earlier conceptualizations of “social imaginaries” (e.g., Taylor 2002, 2004)—a concept that denotes how groups of people collectively imagine their rapidly changing social world and surroundings and how this shapes choices and practices. Landscape imaginaries are embedded in images and stories (e.g., legends). The latter brings it close to the concept of discourses—“a shared way of interpreting the world embedded in language” (Dryzek 2022). Discourses determine how people perceive and frame the world, and therefore also their views on nature, sustainability, biodiversity, and conservation (Soini and Aakkula 2007; Sumares and Fidélis 2011; Walsh 2020). Recognizing that discourses determine how people make sense of environmental problems and solutions, the importance of discourse analysis in nature conservation and landscape governance is broadly acknowledged (Schmidt 2008; Arts and Buizer 2009; Sumares and Fidélis 2011; De Koning et al. 2014; Patrick Bixler et al. 2015; Buizer et al. 2016; Van Assche et al. 2017). Discourse analysis helps explain governance transformations (Van Assche et al. 2017) and changes in policies, programs, and institutions, revealing shifting ideas and norms and ways of speaking and writing about them in the communication among different actors (Schmidt 2008). Discourses shape the narrative upon which people make decisions, and as such, they help explain the successes and failures of conservation initiatives and local buy-in or the absence thereof (Ludwig et al. 2012; Pecurul-Botines et al. 2014). Discourses are, therefore, an intrinsic part of governance; the reason why Buizer et al. (2016) include them in their definition of landscape governance as “the interplay of discourses, institutional practices, and natural-spatial conditions” (p. 448).

Q-methodology explores peoples’ perspectives on issues and can help frame ‘issues’ more robustly through a process of participatory inquiry. It serves both as a *methodology*—an approach to research serving a unique ontological and epistemological calling—and a helpful *method—*a specific tool used to elicit information. It combines qualitative data collection (research participants’ sorting of statements on a given topic according to the degree to which they agree or disagree with them) with subsequent quantitative factor analysis to inductively uncover different perspectives on a social-ecological issue and the extent to which these are shared (and contested) among stakeholders (Webler et al. 2009; Leipold et al. 2019). It has been used to unravel discourses and discourse coalitions related to conservation (Dempsey 2021; Janssens et al. 2022), green economies (D’Amato et al. 2019), ecosystem services management (Grimsrud et al. 2020; Moros et al. 2020), wildlife policies (Ludwig et al. 2012; Holmes et al. 2020), fisheries management (Hiedanpää et al. 2020), plastic waste management (Heath and Cotton 2021), and hydrological fracturing (McLaughlin and Cutts 2018), amongst others. Although identifying “discourse coalitions” (Schmidt 2008; Arts and Buizer 2009) is key for implementing integrated landscape approaches, applying the Q-methodology in such approaches is still largely unexplored (but see Siangulube 2024 and Bayala 2024, this issue).

## Boundary Phenomena

Q-methodology fills an operational and epistemic gap for ILAs (Reed et al. 2020a). By definition, ILAs are *integrative*: they attempt to span boundaries—typically those between siloed governmental bodies, including customary authorities existing in the landscape. Do regional planning agencies, ministries of mining, transport, agriculture, forestry, or conservation agencies interact, cooperate, or understand each other? Similarly, ILAs intend to span sectoral gaps: do estate crop companies manage their concessions in ways that retain complexity in the landscape to deliver the multifunctionality required of them by the range of interested and affected stakeholders? Do introduced commercial entities account for social-environmental harms or other landscape ‘externalities’ resulting from their actions? Do conservation initiatives do more harm than good for wildlife or local people?

Meanwhile, other, less obvious boundaries exist: people imagine their landscapes differently and maintain different views about how nature or landscape contributes to well-being. These underlying differences correlate to different patterns of behaviors in landscapes (van Noordwijk et al. 2023). People will inherently assume different causality for diversely or fuzzily defined change aspirations (who should do what and how to solve collectively ill-defined problems?). A strong diagnosis of these differences involves bringing diverse actors together to think, articulate, and document how they understand and perceive landscape issues. Therefore, Q-methodology has been proposed as a helpful diagnostic and boundary object to begin articulating and bridging divides (Reed et al. 2020c) and has been usefully applied to uncover pathways toward inclusive governance, an overarching objective for ILAs (see the contributions in this collection by Bayala 2024; Dugasseh et al. 2024; Jayaprakash and Hickey 2024; Siangulube 2024; Ng et al. 2023).

**Fig. 1:**
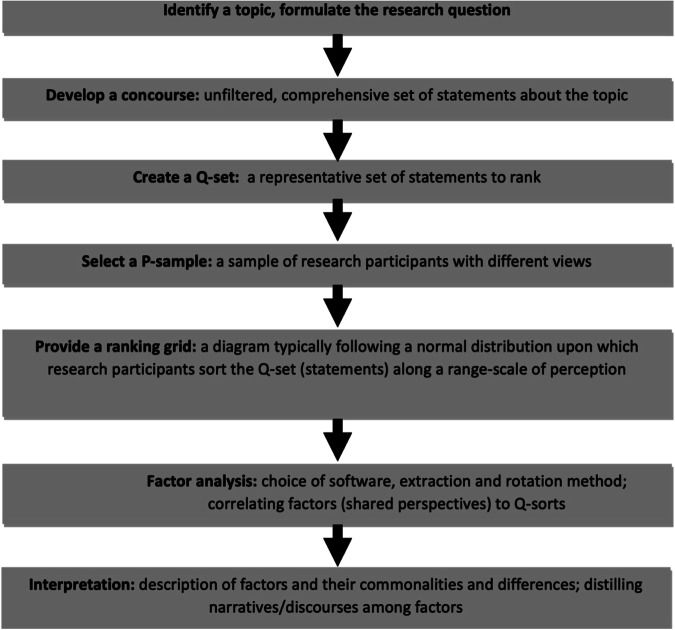
Steps in Q-methodology Source: Adapted from Stenner et al. (2008) and Zabala et al. (2018)

To span ‘landscape imaginary boundaries’ using Q-methodology, the identification of an issue and ‘question to answer’ is as vital as how actors envision solutions. A process of meaningful problem-framing and diagnosis requires thoughtful participation, iteration, and reflexivity across researchers and participants. Transforming the researcher-subject relationship toward a decolonized endeavor, based upon co-learning, co-creation, and reciprocity offers pathways to what business and management studies frame as ‘improved relational capabilities’ (i.e., the capacity to build trust, collaborate with other stakeholders, including local communities, and respect multiple values) (Ngugi et al. 2010; Riggs et al. 2023a). Q-methodology can support this by a participatory uncovering of worldviews and ‘power frames’ inherent in landscape contestation (Larson et al. 2022; Özkaynak et al. 2023). It does so in several steps (Fig. 1), from defining a research question and series of statements (the Q-sort) to interpreting narratives resulting from factor analysis. The challenge, then, is how to articulate these with diverse mental models and sources of information that participants will impart through a Q-sort. Engaging with people meaningfully through discussions, inductive learning, observational and informal settings, and preliminary and post-sorting interviews all help in the development of a comprehensive and accurate concourse (i.e., the pool of statements on an issue representing different perspectives or viewpoints that eventually shape the various discourses).

The Q-method, therefore, has multiple attributes as a powerful tool in the implementation of integrated landscape approaches. First, if co-created through iterative participatory inquiry techniques, Q-method research questions can be meaningful to diverse actors in the landscape, and solution spaces become more transparent and negotiable, reflecting key principles of ILAs (Sayer et al. 2013). Second, spanning discursive boundaries leads to an improved, more comprehensive change logic for ILAs. The Q-method allows actors to engage with, see, and learn from divergent imaginaries, values, and knowledge bases upon which decision-makers can act. By making subjectivities “objective”, translational pathways for change emerge (Brunson and Baker 2016). Third, during a Q-analysis, researchers have the option to consider the traits of the respondents in the discussion of the emergent discourses. This discreet specificity can empower discursively marginalized groups (i.e., traditional knowledge holders) to reveal previously unrecognized information to decision-makers and policymakers, a precondition for improved procedural justice (McGarry et al. 2024). Fourth, another potential discursive benefit of using Q-methodology is that the common yet differentiated perspectives generated are readily presentable in statistically defensible and transparent ways. We postulate that this enhances the legitimacy and potential to gain traction among policymakers and resource managers compared to conventional discourse analysis (i.e., qualitative or critical discourse analysis) (Blommaert and Bulcaen 2000; Fairclough 2013) and other social constructivist approaches. This translational research can help enable transformations by unlocking value-setting processes that shape the value-propositions of landscapes and potential distributive justice outcomes (Riggs et al. 2023b).

The precision and replicability offered by Q-methodology as a landscape diagnostic and boundary object is a useful contribution to how actors can install robust learning mechanisms in landscapes. The increased precision and transparency can shine a light on often overlooked issues such as ‘who values what and how’ and where disconnects fall in the narratives shaping peoples’ perspectives. Pursuing questions such as ‘What change is required’ to achieve common but differentiated goals can highlight potential logical fallacies or synergies. A nuanced understanding of different narratives can enable decision-makers to deliver upon values that are underserved or hitherto unarticulated. Dominant narratives often hide or attempt to homogenize value sets. While dominant narratives are useful insofar as they may support broad social cohesion or can be easily translated into policy responses, they often reduce complexity and reinforce exclusive worldviews and values (Roe 1991). Disempowered or ‘othered’ landscape actors can still exert agency to counter oppressive institutional change logic by leveraging “counterhegemonic discourses” (Arias-Arévalo et al. 2023). Q-methodology can uncover these distributional discourse geographies. In support of ILAs, Q-methodology provides cautionary evidence against the promulgation of win–win’ policy solutions, hindering transformative change by obscuring value conflicts.

## Conclusion

We have described the usefulness of Q-methodology for uncovering discourses, including its potential value as a boundary tool in the diagnosis of ‘landscape’. For the wicked problems of contested landscapes, methodological innovations most fit for purpose are primarily behavioral (Opdam et al. 2015; Turnhout 2024). Q-methodology offers a boundary space through its simple technological requirements to begin fostering deeper inter and intrapersonal reflexivity. As ILAs are ‘process-centric’ and sensitive to justice in its many dimensions, we appreciate Q-methodology for enabling restorative engagement across divides when working in rural landscapes in the Global South. We have found it useful precisely because of its affordability, accessibility, acceptability, and ease of understanding.

This potent combination of qualities increases its utility; boundary tools can be instrumental in reshaping narratives and deepening the leverage space for achieving sustainability transitions (Linnér and Wibeck 2021). Diverse landscape imaginaries of aspirational futures can be mobilized through discursive strategies that contradict mainstream or colonizing economic and environmental development discourses (Coffey 2016). Q-methodology can help fill this function, but there is a need to further develop its empowering potential by using it to identify discourse coalitions and combatting colonial impositions of whose knowledge, visions, and values count.

If Q-methodology is approached with the same ethos as ILAs, as a process-oriented way of muddling through that emphasizes reflexivity, and is untethered to any singular epistemic or normative lens of ‘landscape sustainability’, then the scope opens up to co-create discourses of deliberative foresight (Muiderman et al. 2020, 2023). We then support its use in transitioning toward anticipatory governance of landscapes— which “involves changing short-term decision-making to a longer-term policy vision, including the notion of foresight” (Boyd et al. 2015, p. S153). We also express a moral need to explore ways in which boundary tools such as Q-methodology enable the propagation of narratives that emanate outwards from landscapes, empowering local capabilities against top-down or external interventions that undermine local agency. Behavioral change requires a multi-directional transition, where actors and institutional arrangements reckon with the way their framings of ‘landscape’ impact upon justice and values of nature. The form and function of narratives deserve greater attention as to how Q-methodology may inspire society-wide change full of complex feedback interactions.

## Footnotes

1 This is not to say that commonalities do not occur, specifically in tropical landscapes. Recurring issues include a fragmented governance structure to deal with landscape dynamics and issues, the co-existence and poor coordination of statutory and customary governance systems, poorly funded and equipped institutions, corruption, vulnerability to climate and environmental change, and prevalent poverty. However, we argue that, although lessons can be learned across different contexts, there are no one-size-fits-all solutions. We have argued that lessons best inform ‘how to engage’ (i.e. process) rather than generating transferable outcome-oriented solutions (Langston et al. 2019b).

2 An earlier special issue in this journal addressed the potential of spatial and visualization tools for integrated and inclusive landscape governance (Ros-Tonen and Willemen 2021; Ros-Tonen et al. 2021).
